# Feasibility of and association between plasma total tau with MCI and AD across racial/ethnic groups utilizing multiple datasets

**DOI:** 10.1002/alz.088586

**Published:** 2025-01-09

**Authors:** Archana J McEligot, Cally Xiao, Kevin Cummins, Ioannis Pappas, Arthur W. Toga

**Affiliations:** ^1^ California State University, Fullerton, Fullerton, CA USA; ^2^ Laboratory of Neuro Imaging, Stevens Neuroimaging and Informatics Institute, Keck School of Medicine, University of Southern California, Los Angeles, CA USA

## Abstract

**Background:**

Circulating plasma total tau (t‐tau) concentrations are increasingly being utilized for Alzheimer’s disease (AD) diagnoses. Nonetheless there is a dearth of data on the use of t‐tau in diverse groups, including Hispanic populations who are at elevated risk for AD. Therefore, we investigated the feasibility of biomarker data availability in diverse groups and subsequently assessed the relationship between t‐tau with mild cognitive impairment (MCI) and AD/dementia in Hispanic participants across multiple datasets available via the Global Alzheimer's Association Interactive Network (GAAIN).

**Method:**

Initially, we explored demographic (age, gender, ethnicity), APOEε4 and t‐tau, as well as MCI and AD/dementia data from nine studies (ADNI, HABS‐HD, TARCC, NACC, APOE4, ALFA, ADNI‐ARG, ACE and 10/66) via GAAIN. Multiple logistic regression analyses, adjusted for co‐variates [(age, sex, education, BMI and APOEε4 (for TARCC)], were conducted to investigate the relationship between t‐tau and AD/dementia in non‐Hispanic white (NHW) and Hispanic participants.

**Result:**

Three studies provided biomarker data, and two (HABS‐HD and TARCC) had sufficient Hispanic sample sizes for model estimation (n_ADNI_ = 9; n_HABS‐HD_ = 793; n_TARCC_ = 867). Among NHW participants, plasma t‐tau was associated with AD (TARCC: OR = 7.88, 95% CI = 3.28, 20.8, p < 0.001) and dementia (HABS‐HD: OR = 1.71, 95% CI = 1.17, 2.47, p = 0.005). However, for Hispanic participants, the association between t‐tau and AD (OR = 2.42, 95% CI = 1.38, 4.21, p = 0.002) was weaker (interaction p = 0.03) in TARCC, and no significant relationship was detected with dementia in HABS‐HD.

**Conclusion:**

Increasing tau concentrations by one pg/ml was associated with nearly an eight‐fold higher odds of AD dementia and 71% increase in AD among NHW participants; however, for Hispanic participants, increasing t‐tau was associated with nearly a two and a half‐fold increase in AD only, suggesting that Hispanic participants may potentially be more susceptible to non‐AD dementia. Although plasma biomarker data are available for Hispanic participants, large population‐based studies need to further recruit diverse groups in order to more feasibly study the role of plasma biomarkers in AD and non‐AD dementia and/or etiology in diverse, at‐risk populations.

